# Could serum zonulin be an intestinal permeability marker in diabetes kidney disease?

**DOI:** 10.1371/journal.pone.0253501

**Published:** 2021-06-25

**Authors:** Larissa Salomoni Carpes, Bruna Bellincanta Nicoletto, Luis Henrique Canani, Jakeline Rheinhemer, Daisy Crispim, Gabriela Corrêa Souza

**Affiliations:** 1 Faculty of Medicine, Post Graduate Program in Food, Nutrition and Health, Universidade Federal do Rio Grande do Sul, Porto Alegre, Rio Grande do Sul, Brazil; 2 Life Sciences Knowledge Area, Universidade de Caxias do Sul, Caxias do Sul, Rio Grande do Sul, Brazil; 3 Faculty of Medicine, Post Graduate Program in Medical Sciences: Endocrinology, Universidade Federal do Rio Grande do Sul, Porto Alegre, Rio Grande do Sul, Brazil; 4 Endocrine Division, Hospital de Clínicas de Porto Alegre, Porto Alegre, Brazil; 5 Food and Nutrition Research Center, Hospital de Clínicas de Porto Alegre, Universidade Federal do Rio Grande do Sul, Porto Alegre, Brazil; Universita degli Studi di Perugia, ITALY

## Abstract

Zonulin is a protein associated with the tight junction complex opening at the intestinal epithelium, previously linked to obesity, cardiovascular diseases, type 2 diabetes mellitus (T2DM) and chronic kidney disease (CKD). However, its role in CKD has not been totally elucidated. This study aimed to evaluate zonulin levels in subjects with diabetic kidney disease (DKD). This case-control study included two cases groups: 1) Advanced DKD cases: T2DM patients with estimated glomerular filtration rate (eGFR) <60ml/min/1.73m^2^; 2) Albuminuric T2DM cases: diabetic patients with urinary albumin excretion (UAE) >30mg/g creatinine, but with eGFR>60ml/min/1.73m^2^. Two control groups were also included: 1) T2DM controls: patients with T2DM without impaired kidney function; 2) Non-T2DM controls: subjects without T2DM and normal renal function. Serum levels of zonulin were measured by ELISA. Eighty-six individuals were included. Zonulin levels was different among study groups (P = 0.003). T2DM controls presented higher zonulin levels than non-T2DM controls [(131.35 (83.0–170.5) vs. 87.25 (54.7–111.8), P = 0.018] and advanced DKD cases [63.72 (45.03–106.0); P = 0.007]. Zonulin showed a positive correlation with eGFR (r = 0.222; P = 0.040), total cholesterol (r = 0.299; P = 0.034), LDL (r = 0.258; P = 0.021), and negative with albuminuria (r = -0.243; P = 0.024) and body fat (r = -0.271; P = 0.014). In the multivariate logistic regression analyses, zonulin levels were independently associated to renal outcomes [OR 0.99 (0.98–0.99, P = 0.012)] after 5-year inclusion. In conclusion, increased zonulin levels in patients with TD2M without renal disease suggest an impaired intestinal permeability. Moreover, its association with renal outcomes could indicate its use as a disease monitoring marker. However, the mechanisms behind this association should be better understood.

## Introduction

The intestinal microbiota has been studied in several pathologies, including type 2 diabetes mellitus (T2DM) [1] and chronic kidney disease (CKD) [2]. In both cases, a reduction in intestinal beneficial bacteria and a proliferation of pathogenically bacteria have been suggested, characterizing a condition named dysbiosis [3]. Besides this unbalance in microbiota patients with advanced CKD have an incremental in microbial urease enzyme, which is responsible for transforming excess urea into ammonia in the gut [4].

Both dysbiosis and ammonia accumulation have an impact in intestinal permeability and CKD progression [5,6]. An impaired gut barrier presents a systemic inflammation pattern which allows bacteria translocation and other pathogens into the circulation [7]. Several factors influence the intestinal barrier and permeability, such as microbiota, diet, use of some medications, alcohol consumption, and smoking [8].

Zonulin is a family peptide produced in the intestinal and hepatic cells that regulates a protein complex named tight junctions [9]. Higher levels of zonulin have been associated with increased intestinal permeability since it induces disruption between the junctions in the epithelial intestine cells [10]. Zonulin is mostly stimulated by bacteria and gluten protein (gliadin), making the intestine permeable by opening enterocytes [11].

Studies regarding zonulin as a marker of intestinal permeability have been associated with several diseases [12]. As far as we know, some conditions were linked to increased zonulin levels, representing their associations with an impaired gut permeability [13]. Non-coeliac gluten sensitivity [14], inflammatory bowel syndrome [15], nkylosing spondylitis [16] and arthritis [17] also have been linked to changes in intestinal permeability. Besides that, non-communicable diseases, such as obesity [18] and cardiovascular diseases [19] were correlated to zonulin levels.

T2DM [20] and CKD [21] also have been associated to serum zonulin, but little is known about zonulin and diabetic kidney disease (DKD). Additionally, there are inconsistences according to the studies’ results. Therefore, this study aimed to evaluate zonulin serum levels in DKD patients compared to healthy and diabetic subjects without kidney disease.

## Methods

### Design and patients

The study design was previously described elsewhere [22]. Subjects attending to an Endocrinology outpatient clinic at the Hospital de Clínicas de Porto Alegre were invited to this case-control study. Recruitment date ranged between October 2013 and November 2014 and was based on renal function and T2DM diagnosis. Two case groups were included: 1) Advanced DKD cases: T2DM patients with estimated glomerular filtration rate (eGFR) <60 mL/min/1.73m^2^, and 2) Albuminuric DKD cases: T2DM patients with urinary albumin excretion (UAE) ≥ 30 mg/g creatinine and eGFR ≥60 mL/min/1.73m^2^. Once cases were included, controls were sought based on similar age, gender and body mass index (BMI) and were divided into two control groups: 1) T2DM controls: patients with T2DM with UAE <30 mg/g creatinine and eGFR ≥60 mL/min/1.73m^2^, and 2) Non-T2DM controls: individuals without diabetes and eGFR ≥60 mL/min/1.73m^2^. Exclusion criteria used in the original study were age below 18 years, cancer, pancreatitis, dialysis, acute infection, previous transplantation, pregnancy, and alcohol or drug abuse. The present study also excluded patients with non-authorization data for new studies, with inflammatory bowel diseases and those with serum zonulin greater than 400ug/dL (because limitation in the assay).

Diabetes was defined according to American Diabetes Association criteria [23] and renal function was estimated using the Chronic Kidney Disease–Epidemiology Collaboration (CKD-EPI) formula [24]. The study was approved by the Ethics Committee of the Hospital de Clínicas de Porto Alegre, and all subjects gave informed written consent before participation.

### Clinical, anthropometric and biochemical data evaluation

Clinical and sociodemographic data were collected through questionnaires and electronical charts, and included age, gender, ethnicity, T2DM duration, hypertension, and medication use.

Anthropometric evaluation consisted of weight and height measured in anthropometric scale with a stadiometer, for later body mass index (BMI) estimation. Body composition was evaluated by an electric bioimpedance (*InBody 230; Biospace*, Seul, Korea). All measures were made while fasting, light clothes and without shoes [25].

Blood and spot urine tests were performed after 12-hour overnight fast. Levels of fasting plasma glucose (Hexokinase UV enzymatic), HbA1c (HPLC), total cholesterol (enzymatic colorimetric), HDL-cholesterol (homogeneous enzymatic colorimetric), triglycerides (TG) (enzymatic colorimetric), high-sensitivity C-reactive protein (hsCRP) (immunoturbidimetry), albuminuria (immunoturbidimetry), and serum creatinine (Jaffe method) were determined using standardized techniques. LDL-cholesterol was calculated according to Fridelwald formula: LDL-c = total cholesterol–(HDL-cholesterol + triglycerides / 5), when TG was below 400 mg/mL.

Blood samples were collected, centrifuged and kept in storage at -80°C for zonulin and interleukin-6 (IL-6) analysis. Zonulin concentration was quantified in serum samples, using the haptoglobin (encoded HP; Hp2-Alpha; Alpha-2-Macroglobulin; Zonulin) concentration assessed by ELISA (Cloud-Clone Corp, Katy, TX). The assay sensitivity was 2.92 ng/mL and assay range was 6.25–400 ng/mL, while inter-assay variability was less than 12% for serum samples. All zonulin samples were analyzed in duplicates. IL-6 concentration was determined in serum samples, using Human IL-6 Quantikine ELISA kit (R&D Systems, Minneapolis, MN, USA). Only 30.7% samples were analyzed in duplicates.

After 5 years of study inclusion, outcomes of each patient were assessed through medical online records or by phone call when no information was available. The evaluated outcomes were: decline in renal function (defined by change in stage of CKD: yes or no), onset dialysis or renal transplantation, stroke, myocardial infarction, coronary procedures (percutaneous coronary intervention and myocardial revascularization surgery), and death.

### Statistical analysis

Data were analyzed through the Statistical Package for Social Sciences, version 18.0 (SPSS Inc, Chicago, IL). Continuous variables were tested for normality by Shapiro-Wilk test. Since zonulin concentrations presented asymmetric distribution, this variable was logarithmically transformed before analysis. Comparisons between groups were performed using Chi-Square, One-Way ANOVA with Levene and Tukey or Kruskal-Wallis with Dunn tests, when appropriate. Correlations were tested by Pearson’s or Spearman’s correlation coefficient, according to variable distribution. Significant variables from the correlations and that were not used in T2DM and CKD diagnosis were applied to correct zonulin levels by a regression model. These variables were LDL and body fat percentage. Then, adjusted zonulin levels were compared among groups using ANCOVA with Bonferroni test. For this analysis, zonulin log values were used, but data presented in the result section are described as median for better understanding of the findings. Zonulin levels were compared between patients who have or not have outcomes at 5 years after study inclusion through Student’s t test. Multivariable Poisson regression analysis was performed to identify the association of zonulin and presence of renal outcomes, adjusted for T2DM, dyslipidemia, smoking, obesity and age, known as possible risk factors to higher intestinal permeability [13]. The level of statistical significance was established as 5%.

Sample size calculation was based on a previous study [26] considering a standard deviation = 4, size effect = 0.54, power of 95% and level of significance of 5%. The total sample estimated was 56 subjects. Because greater sample size was available from the previous study, we had a power of 99.6%.

## Results

From 114 eligible patients from the previous study [22], 28 were excluded because their serum zonulin levels were greater than 400ug/dL or they did not consent to the new study, resulting in 86 patients included in the present analysis.

The total sample was composed mostly by women (57%), with white ethnicity (67.4%) and the mean age was 61.2 ± 1.02 years. Sociodemographic and clinical data among study groups are presented in Table 1. Age, gender, ethnicity, smoking, BMI, body fat, total cholesterol, and hsCRP were similar between groups. T2DM duration was also similar between groups with diabetes. Advanced DKD cases presented worst HDL-cholesterol, triglycerides, and IL-6 levels. Non-T2DM controls had lower hypertension prevalence and higher LDL-cholesterol levels (Table 1).

**Table 1:** Sociodemographic, clinical, biochemical and anthropometric characteristics among groups.

	Non-T2DM control (n = 18)	T2DM control (n = 26)	T2DM albuminuric (n = 20)	DKD (n = 22)	P value
Age (years)	59.50 ± 10.37	59.73 ± 9.15	64.05 ± 8.63	61.68 ± 9.99	0.391
Female, n (%)	11 (61.1)	14 (53.8)	13 (65.0)	11 (50.0)	0.755
Caucasian, n (%)	11 (61.1)	18 (69.2)	15 (75)	14 (63.6)	0.808
Non-smoker, n (%)	9 (50.0)	14 (53.8)	9 (45.0)	13 (59.1)	0.540
T2DM duration (years)	-	15.57 ± 9.49	14.3 ± 7.98	18.22 ± 9.24	0.353
Hypertension, n (%)	5 (33.3)^a^	25 (96.2)^b^	20 (100)^b^	22 (100)^b^	<0.001
eGFR (mL/min/1,73m^2^)	100.0 (88.45–114.25)^a^	95.1 (85.38–116.25)^a^	98.0 (92.0–106.0)^a^	23.0 (17.0–33.64)^b^	<0.001
Albuminuria (mg/L)	7.40 (2.99–12.30)^a^	11.70 (4.65–19.95)^a^	98.70 (61.30–209.97) ^b^	618.15 (200.15–1854.7) ^b^	<0.001
Serum creatinine (mg/dL)	0.69 (0.58–0.87) ^a^	0.73 (0.60–0.95) ^a^	0.72 (0.55–0.84) ^a^	2.51 (2.02–3.29) ^b^	<0.001
Fasting glucose (mg/dL)	90.0 (83.0–94.0) ^a^	140.0 (113.75–169.50) ^b^	162.0 (88.25–191.25) ^b^	126.0 (73.0–171.5) ^b^	<0.001
HbA1c (%)	5.5 (5.3–5.7) ^a^	7.85 (7.0–9.12) ^b^	8.7 (7.6–9.4) ^b^	7.9 (7.2–9.4) ^b^	<0.001
Total Cholesterol (mg/dL)	200.28 ± 33.08	179.15 ± 48.28	179.40 ± 40.52	181.73 ± 53.03	0.413
HDL-Cholesterol (mg/dL)	45.67 ± 8.97 ^a^	40.65 ± 7.71 ^a^	38.75 ± 11.0 ^a^	36.5 ± 10.48 ^b^	0.026
LDL-Cholesterol (mg/dL)	125.8 (101.95–144.3) ^a^	97.7 (72.4–136.1) ^ab^	94.0 (84.85–99.9) ^b^	89.0 (72.0–122.5) ^b^	0.008
Triglycerides (mg/dL)	126.56 ± 51.55 ^a^	156.69 ± 69.78 ^ab^	253.05 ± 213.10 ^b^	255.64 ± 177.42 ^b^	0.007
hsCRP (mg/dL)	3.06 (1.72–9.29)	3.44 (1.36–8.01)	2.66 (1.75–4.28)	4.54 (1.81–17.52)	0.262
IL-6 (pg/dL)	3.12 (3.12–3.18) ^a^	3.12 (3.12–3.5) ^a^	3.12 (3.12–4.06) ^a^	7.03 (3.94–9.66) ^b^	<0.001
BMI (kg/m^2^)	28.2 (25.5–33.0)	30.0 (26.3–32.8)	31.7 (27.7–38.1)	30.9 (28.0–38.5)	0.078
Body fat (%)	37.1 (29.5–43.5)	34.8 (28.0–42.7)	38.35 (23.7–46.1)	38.25 (27.9–47.2)	0.700

Data expressed in n (%), median ± SD or median and interquartile range (P25 –P75). Different letters means p <0.05. T2DM: Type 2 diabetes mellitus; HbA1c: Glycated hemoglobin; IL-6: Interleukin-6; BMI: Body mass index; hsCRP: High sensitivity C-reactive protein; eGFR: Estimated glomerular filtration rate.

**Fig 1:**
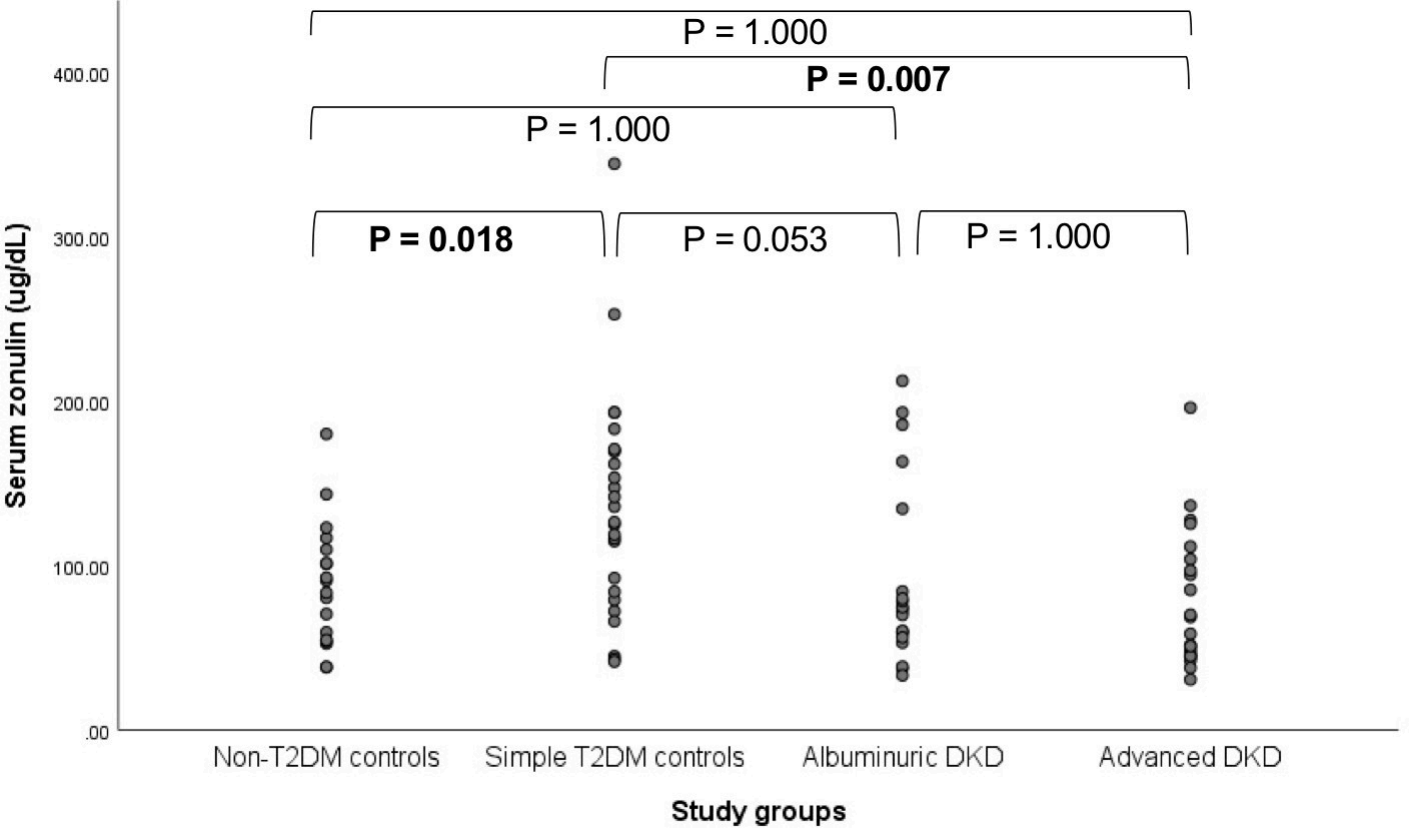
Serum zonulin levels among groups. Serum zonulin levels are expressed in median among different study groups. Values in bold indicate p<0.05. T2DM: Type 2 diabetes mellitus; DKD: Diabetic kidney disease.

Serum zonulin levels were significantly different among groups (P = 0.003) and are depicted in Fig 1. T2DM controls showed higher zonulin levels than non-T2DM controls [(131.35 (83.0–170.5) vs. 87.25 (54.7–111.8); P = 0.018] and advanced DKD cases [63.72 (45.03–106.0); P = 0.007].

Zonulin presented a positive correlation with eGFR (r = 0.222; P = 0.040), total cholesterol (r = 0.299; P = 0.034), LDL (r = 0.258; P = 0.021), and negative with albuminuria (r = -0.243; P = 0.024) and body fat (r = -0.271; P = 0.014) (Table 2).

**Table 2:** Correlations between serum zonulin and renal, glycemic, lipid, inflammatory and body composition parameters.

	R (p)
	N = 86
eGFR (ml/min/1.73 m^2^)	**0.222 (0.040)**
Creatinine (mg/dL)	-0.171 (0.116)
Albuminuria (mg/L)	**-0.243 (0.024)**
Fasting glucose (mg/dL)	0.136 (0.211)
HbA1c (%)	0.045 (0.681)
HOMA-IR	-0.018 (0.873)
Total Cholesterol (mg/dL)	**0.229 (0.034)**
HDL-Cholesterol (mg/dL)	-0.117 (0.285)
LDL-Cholesterol (mg/dL)	**0.258 (0.021)**
Triglycerides (mg/dL)	0.102 (0.384)
IL-6 (pg/mL)	-0.180 (0.099)
hsCRP (mg/dL)	-0.135 (0.220)
BMI (kg/m^2^)	-0.198 (0.067)
Body fat (%)	**-0.271 (0.014)**
Lean mass (kg)	0.182 (0.102)
Free fat mass (kg)	0.178 (0.109)
Total body water (kg)	0.173 (0.121)

BMI: Body mass index; DM: Diabetes mellitus; eGFR: Estimated glomerular filtration rate; HbA1c: Glycated hemoglobin; hsCRP: High sensitivity C-reactive protein; IL-6: Interleukin-6.

After 5 years, 66 patients (76.7% of the sample) were evaluated for renal and cardiovascular outcomes and death. With respect to renal outcomes, 24 patients had renal function decline (39.3%), 10 patients started dialysis (15.5%) and just one patient had renal transplantation (1.5%). Regarding cardiovascular outcomes, 6 patients had heart attack (9.2%), 3 patients had stroke (4.6%), 7 patients performed any cardiovascular surgery (10.8%) and 9 patients died (13.8%).

Patients who presented renal outcomes had lower zonulin levels (P = 0.007), as shown in Table 3. In the multivariate logistic regression analysis, zonulin levels [OR 0.99 (0.98–0.99, P = 0.012)] and hypertension [OR 0.07 (0.01–0.62, P = 0.017)] were independently associated to renal outcomes, after adjustment for T2DM, dyslipidemia, smoking, obesity, and age.

**Table 3:** Association between zonulin (log) and renal, cardiovascular and death outcomes.

Patients (n = 65)	Zonulin	p
Renal outcomes		**0.007**
Yes (n = 30)	4.27 ± 0.50	
No	4.64 ± 0.57	
Cardiovascular outcomes		0.204
Yes (n = 16)	4.31 ± 0.47	
No	4.52 ± 0.59	
Death		0.060
Yes (n = 9)	4.13 ± 0.40	
No	4.52 ± 0.58	

Values are expressed in mean ± standard deviation.

## Discussion

In the present study, patients with T2DM and normal renal function presented elevated levels of zonulin. On the other hand, patients with compromised renal function, represented by eGFR < 60mL/min/1.73m^2^, had lower zonulin levels. Correlations of zonulin with renal function, body fat percentage and lipid profile were also found in the present study.

It has been documented that T2DM patients present higher zonulin levels than non-diabetic subjects [20,26–28]. This evidence was observed in different stages of the disease. Even in pre-diabetes condition, zonulin concentration were reported to be increased [27]. In newly-diagnosed [20] and longstanding T2DM patients [26], zonulin levels were also elevated, when compared to healthy subjects. The zonulin increment may indicates a higher intestinal permeability in T2DM, since previous evidence describes zonulin as intestinal permeability marker in diabetes [29].

However, the role of zonulin as a marker of intestinal permeability in kidney disease is controversial. In the present study, lower zonulin was found in advanced DKD patients when compared to T2DM controls, but values were similar to the non-T2DM group. Moreover, when eGFR decreases, the zonulin also decreases, as demonstrated by the positive correlation between these parameters. This finding was different than expected. The main hypothesis of this study was that serum zonulin could be a marker of intestinal permeability in DKD, being elevated in this condition, since CKD presents permeability impairment [30]. However, previous studies [21,31–34] present distinct results, in both directions.

Similar to our findings, Hasslacher et al. [26] reported a positive correlation between zonulin levels and eGFR and a negative one with albuminuria in T2DM patients. The positive association between zonulin and eGFR were also observed in a sample of heart failure patients [35], reinforcing that the decrease in renal function is followed by a decrease in serum zonulin. In addition, there are evidences that pre-dialysis [21] and post-renal transplantation patients [31] have lower zonulin levels than controls. Some authors suggest that zonulin may be depleted in the kidneys due to renal failure [26,35].

On the other hand, there are also evidence of a negative correlation between zonulin and eGFR, demonstrating that when renal function declines, zonulin could increase [33]. Moreover, a study with T2DM with advanced renal disease found higher zonulin levels than controls, as well as lipopolysaccharide (LPS) and trimethylamine-N-Oxide (TMAO) [34]. In hemodialysis patients, zonulin levels were higher than healthy individuals [32] and pre-dialysis patients [33]. In this case, it was hypothesized that less renal elimination increases serum zonulin [32].

It is suggested that zonulin may have other function in CKD, that not intestinal permeability. A systematic review showed conflicting results with respect to permeability assessment methods in CKD. The authors conclude that CKD patients have impaired intestinal permeability; however, data interpretation should be cautious, and new markers which do not have renal influence or gut bacteria are necessary [36]. In the same way, it was previously documented that constipation is associated with increased uremic toxins that impacts on intestinal permeability [37]. However, zonulin levels were not different among peritoneal dialysis patients according to the presence of constipation [38]. Moreover, after an intervention with prebiotics, zonulin levels did not change despite the reduction of the p-cresyl uremic toxin [39]. These findings are in agreement with the hypothesis that zonulin may have other function in CKD.

In the present study, lower zonulin concentrations were observed in patients who presented renal outcomes, such as renal function decline and hemodialysis after 5 years study inclusion. Moreover, zonulin levels were an independently preditor of renal outcomes. A hypothesis for the possible mechanism is the increase in renal glomerular permeability, with increase in albuminuria, allowing zonulin entrance, and impacting in renal health. It is in agreement with the negative correlation found in this study between zonulin and albuminuria.

In this context, zonulin could be a predictor of DKD progression and not an intestinal permeability marker. Poor outcomes were previously associated to lower zonulin levels in other conditions. A study evaluating intestine integrity in HIV patients showed that higher zonulin levels seem to predict survival [40]. Still, when measuring cardiovascular and all causes mortality related markers, a study with heart failure patients demonstrated that lower zonulin levels could point to worst prognosis [35].

In obesity, zonulin has been studied as a marker of intestinal permeability [18]. Some studies observed higher zonulin levels in obese patients, according to body mass index [18,20,41]. Even so, its increment could be an independent risk factor for overweight and obesity [42]. Nonetheless, data evaluating zonulin levels and body composition is scarce. Our findings show a controversial association, where higher zonulin levels were correlated to less body fat. However, this data should be carefully interpretated, considering that our sample was mainly represented by DKD patients.

According to serum lipid evaluation, there was also a positive correlation between zonulin and total cholesterol and LDL, which is in agreement to the literature. Previous studies reported the association of zonulin with triglyceride and total cholesterol [20,41]. Moreover, higher zonulin levels were also associated with hyperlipidemia [42]. This association could be explained due to the communication between the gut and adipose tissue [43].

This study has some limitations, such as the lack about urinary zonulin values. Furthermore, the sample size is relatively small, however we had power for study conduction, based on previous sample size calculation.

This is the first study assessing serum zonulin levels in renal complications of T2DM in Brazilian subjects, with evaluation of major outcomes. Serum zonulin seems to be an impaired intestinal permeability marker in T2DM patients with normal renal function. Nevertheless, DKD impact in zonulin levels is not totally elucidated. Further studies are necessary to investigate the association of zonulin in DKD and explore its use as a marker of intestinal permeability or the disease progression. In addition, we suggest different intestinal permeability assessment markers in DKD, for further clarification.

## Supporting information

S1 Data (SAV)
